# Reflection on modern methods: calculating a sample size for a repeatability sub-study to correct for measurement error in a single continuous exposure

**DOI:** 10.1093/ije/dyz055

**Published:** 2019-04-23

**Authors:** Katy E Morgan, Sarah Cook, David A Leon, Chris Frost

**Affiliations:** 1 Faculty of Epidemiology and Population Health, London School of Hygiene & Tropical Medicine, London, UK; 2 Department of Community Medicine, UiT The Arctic University of Norway, Tromsø, Norway

**Keywords:** Measurement error, regression dilution bias, repeatability, reliability, sample size

## Abstract

Using a continuous exposure variable that is measured with random error in a univariable linear regression model leads to regression dilution bias: the observed association between the exposure and outcome is smaller than it would be if the true value of the exposure could be used. A repeatability sub-study, where a sample of study participants have their data measured again, can be used to correct for this bias. It is important to perform a sample size calculation for such a sub-study, to ensure that correction factors can be estimated with sufficient precision. We describe how a previously published method can be used to calculate the sample size from the anticipated size of the correction factor and its desired precision, and demonstrate this approach using the example of the cross-sectional studies conducted as part of the International Project on Cardiovascular Disease in Russia study. We also provide correction factors calculated from repeat data from the UK Biobank study, which can be used to help plan future repeatability studies.

**Key Messages**

- Measurement error in a single continuous exposure variable leads to regression dilution bias when estimating associations with other variables.
- This bias can be corrected for using data from a repeatability sub-study, where a subset of the main study participants are re-measured.
- It is advisable to perform a sample size calculation for such a sub-study, to ensure that correction factors are estimated with sufficient precision.
- Sample size calculations can be made using a previously published approach that requires the expected size of the correction factor as well as its desired precision.
- Estimates from UK Biobank data can be used to determine the size of correction factors that one may expect for different types of exposure.

## Introduction

Continuous clinical data in epidemiological studies are often collected with some measurement error,^1–4^ with the value recorded for any one individual differing from their true underlying value. One possibility is that this measurement error is essentially random, being unrelated to the true underlying level of the variable in question or any other variable. Such measurement error can be caused by day-to-day fluctuations or by imprecision introduced by the equipment used to make measurements, and can effectively be regarded as being generated by a random process. When using a continuous measurement that is subject to non-differential random error as a predictor in a univariable regression model, the regression slope obtained will, in expectation, be smaller in magnitude than if the true value were used. This is known as regression dilution bias,^5^ or as attenuation or bias towards the null. One way to correct for this bias is to collect two measurements on a sub-sample of people, and use these repeats to calculate a correction factor for the regression slope.

When planning a repeatability sub-study (sometimes referred to as a reliability study), it is necessary to decide how many people to re-measure. This will be partially driven by logistics: collecting data is time consuming and expensive. However, if too few people are re-measured, correction factors will be imprecisely estimated and corrected regression coefficients will have wide confidence intervals (CIs). It is therefore advisable to perform a sample size calculation for a repeatability sub-study before collecting extra data.

There is a literature on sample sizes for repeatability studies, including examples listed.^6–11^ In this paper, we follow the approach outlined by Giraudeau and Mary.^9^ We provide a practical guide on choosing the sample size for a repeatability sub-study. We give a basic introduction to measurement error, demonstrate how to calculate a sample size, give an example of a repeatability sub-study from the International Project on Cardiovascular Disease in Russia (IPCDR)^12^ and provide several correction factors estimated from UK Biobank data.^13^

## Measurement error and regression dilution bias

Consider a simple setting where a continuous outcome $Y_{i}$ from person i has a linear relationship with a continuous variable $X_{i}$, the true exposure. Ideally, we would like to obtain an estimate of $\beta_{X}$ from the regression model:

(1) $$Y_{i}=\alpha_{X}+\beta_{X}X_{i}+\epsilon_{X,i}$$

However, suppose we are only able to measure $W_{\mathrm{ij}}$, a variable measured with non-differential error at one particular occasion j, not $X_{i}$. In the classical measurement error model, error is considered to be random conditional on the true value and uncorrelated between repeated measurements. We can express this relationship between $W_{\mathrm{ij}}$ and $X_{i}$ algebraically as:

(2) $$W_{\mathrm{ij}}=X_{i}+\epsilon_{\mathrm{ij}}$$

where $\epsilon_{\mathrm{ij}}$ is the random error. This error is assumed to be independently normally distributed: $\epsilon_{\mathrm{ij}}∼\mathrm{NID}(0,\;\sigma_{\epsilon }^{2})$: there is no systematic bias (since the mean is zero), the errors have constant variance and are independent of each other and of $X_{i}$.

If we simply use $W_{\mathrm{ij}}$ in our regression model:

(3) $$Y_{i}=\alpha_{W}+\beta_{W}W_{\mathrm{ij}}+\epsilon_{W,i}$$

it can be shown that $\left|\beta_{W}\right|<\left|\beta_{X}\right|$,^3^,^5^ i.e. that in truth the regression coefficient relating to the error-prone measurement will be smaller in magnitude than that for the true error-free exposure, and specifically that:

(4) $$\beta_{W}=\rho \beta_{X}\;\mathrm{where}\;\rho =\frac{\sigma_{X}^{2}}{\sigma_{X}^{2}+\sigma_{\epsilon }^{2}}$$

Here $\sigma_{X}^{2}$ is the variance of $X_{i}$ and $\sigma_{\epsilon }^{2}$ is the measurement error variance, implying that the variance of $W_{\mathrm{ij}}$ is $\sigma_{X}^{2}+\sigma_{\epsilon }^{2}$. The ratio of the variance of $X_{i}$ to that of $W_{i}$ is the intracluster correlation coefficient (ICC), ρ. In order to get an estimate of $\beta_{X}$ we would have to multiply our estimated slope ($\hat{\beta_{W}}$) by the reciprocal of an estimate of the ICC. We term the true value of this correction factor λ.

In the absence of a ‘gold-standard’ measure of $X_{i}$, λ can be estimated by taking one further measurement on a sub-set of participants and fitting a standard random intercepts linear mixed effects model to the data to obtain estimates of$\;\sigma_{X}^{2}$ and $\sigma_{\epsilon }^{2}$. Since λ is estimated from observed data there will be some uncertainty associated with it, which will depend partly on the size of the sub-set. If an estimate of $\beta_{X}$ is made from the observed regression coefficient$\hat{{\;\beta }_{W}}\;$and the correction factor $\hat{\lambda },\;$then the uncertainty in both these estimates needs to be taken into account when calculating a confidence interval for $\beta_{X}$.^14^ In the next section we explain how to calculate a sample size for such a repeatability sub-study.

Here, we have focused on a single continuous error-prone exposure variable that has a linear relationship with the outcome. Correcting for measurement error in other situations is more complex. For example, in multiple regression when there is more than one error-prone predictor it is possible for measurement error to increase one or more of the regression coefficients, and correcting for measurement error becomes more complex. Estimates have to be made of both the between- and the within-person variances for each predictor, and also of the correlations between the predictors, in order to be able to make appropriate corrections.^15^ In general estimating sample sizes for a multiple regression analysis can be challenging, since researchers may not have estimates of all the variances and correlations. In such situations, one approach would be to base the sample size on the ICC for the most error prone measure. However, assessing the performance of such a simplifying approach is beyond the scope of this paper.

There is one situation where the correction methodology for simple linear regression can be extended to multiple regression in a simple fashion: where there is one error-prone predictor and all the other covariates are error-free. In this situation, there is still attenuation of the regression coefficient for the error-prone predictor, and the correction factor becomes that given in Equation (4) but with the unconditional variances replaced with variances that are conditional on the error-free covariates.^2^ In such a case, for example where there is an error-prone variable and the regression model of interest adjusts for age and sex (which can often be considered to be measured without error), the methods in this paper can still be used but substituting conditional variances where appropriate. These conditional variances can be estimated by fitting the same random intercepts model as above, but incorporating age and sex as fixed covariates.

## Sample size calculations for a repeatability sub-study

Giraudeau and Mary^9^ suggest basing the sample size of the repeatability study on the width of the 95% CI for the intraclass correlation coefficient (ICC), where the correction factor is the inverse of the ICC. We slightly modify this approach, basing the sample size on the width of the 95% CI around the estimated correction factor $\hat{\lambda }$. For example, we might wish to have a 95% confidence interval that extends from 10% below the estimate to 10% above the estimate (we denote this using parameter δ=0.1) or from 20% below to 20% above the estimate (δ=0.2). $\hat{\lambda }$ is unknown at the design stage but provided we have a “planning value” $\bar{\lambda }$ (using terminology and notation analogous to that in Shoukri *et al*.^10^) we can use this to calculate a sample size as follows.

An estimate of λ can be obtained from:

(5) $$\hat{\lambda }=\frac{{\hat{\sigma }}_{X}^{2}+{\hat{\sigma }}_{\epsilon }^{2}}{{\hat{\sigma }}_{X}^{2}}\;,$$

where the hats indicate that these are estimates from our observed validation dataset. An approximate variance formula^14^ for the estimated correction factor is:

(6) $$V\left(\hat{\lambda }\right)\approx \frac{{\left(\lambda^{2}-1\right)}^{2}}{n}\;.$$

where n is the number of participants with repeated measures. This demonstrates that, in approximation, the variance of the estimate of the correction factor only depends on λ and n.

Using the standard normal approximation for a 95% confidence interval, with $\overset{}{\hat{\lambda }}$ replaced by the planning value $\bar{\lambda }$, it follows that n is given by:

(7) $$n={\left(\frac{1.96}{\delta }\right)}^{2}{\left(\frac{{\bar{\lambda }}^{2}-1}{\bar{\lambda }}\right)}^{2}$$

For example, consider a repeatability sub-study for a variable that is expected to have a correction factor of about 1.5. Suppose that the researchers want to be able to estimate the correction factor with a precision of $\bar{\lambda }\pm 0.2\bar{\lambda }$, such that δ=0.2 and the 95% CI would span 1.2 to 1.8. The sample size can be calculated as follows:

(8) $$n={\left(\frac{1.96}{0.2}\right)}^{2}{\left(\frac{1.5^{2}-1}{1.5}\right)}^{2}=66.7$$

A repeatability sub-study of 67 people would therefore give the desired precision, assuming that the correction factor is estimated to be approximately 1.5.

A range of sample sizes for different expected correction factors and CI widths can be calculated using Equation (7) and summarised in a table. An example is given in Table 1.

**Table 1.** Sample sizes required in the repeatability sub-sample for different planning values of the correction factor $\bar{\lambda }$ and different 95% confidence intervals

$\bar{\lambda }$	$\bar{\lambda }\pm 0.1\bar{\lambda }$	$\bar{\lambda }\pm 0.2\bar{\lambda }$	$\bar{\lambda }\pm 0.3\bar{\lambda }$
1.1	−^a^	−^a^	−^a^
1.2	52	−^a^	−^a^
1.3	108	27	−^a^
1.4	181	45	−^a^
1.5	267	67	30
1.6	365	91	41
1.7	475	119	53
1.8	595	149	66
1.9	725	181	81
2.0	864	216	96
2.25	1252	313	139
2.5	1694	424	188
2.75	2188	547	243
3.0	2732	683	304
3.5	3969	992	441
4.0	5402	1351	600

a These entries are left blank since the 95% CIs for these values would include 1. Since, in truth, correction factors cannot be less than one, this is indicative of the fact that the sample sizes here are too small for the large sample approximations used in calculating the CIs to be reliable.

## Example: the International Project on Cardiovascular Disease in Russia

The International Project on Cardiovascular Disease in Russia (IPCDR) is a large, multi-method study looking at the reasons for extremely high cardiovascular disease mortality in Russia. One major component of IPCDR is a large cross-sectional study^12^ conducted in two Russian cities, Novosibirsk and Arkhangelsk (2015–18), including a baseline interview completed in participants’ homes by a trained interviewer and a health check at a polyclinic completed by medical professionals. The health check included a variety of physical measurements such as blood pressure, waist and hip circumference and grip strength. Blood samples were also collected. In total 5129 men and women aged 35 to 69 years completed the baseline interview, of whom 4551 also attended the medical examination. In order to address measurement error issues in the cross-sectional study, IPCDR included a repeatability sub-study, and Table 1 was used to choose the sample size for this.

From Table 1, a sample size of 200 people will offer moderate precision on a correction factor of 2 (δ=0.2, giving a 95% CI that has a total width of approximately 40% of the size of the correction factor), higher precision of δ=0.1 on correction factors of around 1.4 and lower precision of δ=0.3 on larger correction factors of around 2.5. Since the measurement error at the two cities may be different, it was decided to recruit 200 people at each. Participants were invited back approximately 1 year after their first health check, to minimize any seasonal effects.

## UK Biobank

One issue when calculating the sample size for a repeatability sub-study is knowing in advance how large correction factors are likely to be. UK Biobank^13^ is a very large study conducted in the UK, consisting of a rich selection of baseline health data on approximately 500 000 participants. Approximately 20 000 people from the baseline assessment took part in a repeatability sub-study that occurred several years after initial measurement. This allowed us to estimate several correction factors for this study which used highly standardized procedures.

Although exact correction factors will vary between studies, depending on factors such as operating procedures and study populations, these UK Biobank estimates could be used as ball-park figures to inform sample size calculations for other repeatability studies. When planning a repeatability study, researchers could use Table 1 to see how the necessary sample size will vary with the correction factor and its required precision. They could then use the UK Biobank estimates to get an idea of how big the correction factor is likely to be for their variables of interest, as well as looking for other published correction factors in the literature, paying attention to how similar the data collection processes are likely to be in their study to try and ensure that the final choice of sample size is driven by the specifics of their own study.

The estimates of correction factors for a range of UK Biobank variables, calculated from 20 346 participants with repeat visit data, are given in Table 2. Mean age at the baseline visit was 57.1 [standard deviation (SD) 7.4, range 40 to 73] years, and 51.2% were female. Baseline visits were conducted between 2006 and 2010, with repeat visits occurring between 2012 and 2013. The mean time between the two visits was 4.3 (SD 0.9, range 2.1 to 7.0) years.

**Table 2.** Estimates of correction factors for a range of variables from the UK Biobank

Variable	Measurement device used	Number of people	Correction factor (95% CI)
Body mass index (BMI)	Constructed from height (Seca 202 height measure) and weight (see below)	20 262	1.08 (1.08 to 1.08)
Weight	Tanita BC-418 MA body composition analyser		
All subjects		20 274	1.05 (1.05 to 1.05)
Restricted to those with weight reported as being about the same as the previous year		11 683	1.03 (1.03 to 1.03)
Waist circumference	Wessex non-stretchable sprung tape measure	20 299	1.17 (1.16 to 1.17)
Hip circumference	Wessex non-stretchable sprung tape measure	20 297	1.21 (1.21 to 1.22)
Fat percentage	Tanita BC-418 MA body composition analyser	19 757	1.09 (1.08 to 1.09)
Whole body fat mass	Tanita BC-418 MA body composition analyser	19 733	1.10 (1.10 to 1.11)
Basal metabolic rate	Tanita BC-418 MA body composition analyser	19 772	1.03 (1.02 to 1.03)
Diastolic blood pressure	Omron HEM-7015IT digital blood pressure monitor		
First reading		19 350	1.64 (1.61 to 1.66)
Second reading		18 917	1.73 (1.70 to 1.76)
Average		18 624	1.58 (1.55 to 1.60)
Systolic blood pressure	Omron HEM-7015IT digital blood pressure monitor		
First reading		19 346	1.55 (1.53 to 1.57)
Second reading		18 916	1.62 (1.60 to 1.64)
Average		18 620	1.50 (1.48 to 1.51)
Pulse rate	Omron HEM-7015IT digital blood pressure monitor		
First reading		19 350	1.61 (1.59 to 1.63)
Second reading		18 917	1.61 (1.59 to 1.64)
Average		18 624	1.56 (1.54 to 1.58)
Pulse rate	Pulse Trace PCA2	4690	1.61 (1.56 to 1.65)
Forced vital capacity^a^	Vitalograph Pneumotrac 6800	16 589	1.22 (1.21 to 1.22)
Forced expiratory volume^a^	Vitalograph Pneumotrac 6800	16 589	1.28 (1.27 to 1.29)
Peak expiratory flow^a^	Vitalograph Pneumotrac 6800	16 589	1.67 (1.65 to 1.70)
Bone mineral density (left heel)	(Sahara Clinical Bone Sonometer)	4407	1.27 (1.25 to 1.29)
Bone mineral density (right heel)	(Sahara Clinical Bone Sonometer)	4430	1.24 (1.22 to 1.25)
Grip strength (left hand)	Jamar J00105 hydraulic hand dynamometer	20 147	1.51 (1.49 to 1.53)
Grip strength (right hand)	Jamar J00105 hydraulic hand dynamometer	20 162	1.57 (1.55 to 1.59)
White blood cell count	Beckman Coulter LH750 Haematology Analyser	18 383	1.38 (1.37 to 1.40)
Haematocrit	Beckman Coulter LH750 Haematology Analyser	18 385	1.39 (1.38 to 1.40)
Mean corpuscular volume	Beckman Coulter LH750 Haematology Analyser	18 384	1.37 (1.36 to 1.38)
Platelet count	Beckman Coulter LH750 Haematology Analyser	18 385	1.39 (1.37 to 1.40)

a Average of two closest readings.

All estimated correction factors are less than 2, but there is a reasonable amount of variation in size between the different variables. For example, BMI and weight have correction factors that are very close to 1, whereas peak expiratory flow and blood pressure have much higher correction factors, implying as might be expected that there is more measurement error in these variables.

In addition to measuring weight, participants were asked whether they weighed more, less or about the same as the previous year. The correction factor for weight, calculated from only those people who said at the repeat visit they weighed about the same as a year ago, is smaller than when not making this restriction (1.03 vs 1.05). This suggests that in the larger sample, some actual change in weight is contributing to the correction factor in addition to any measurement error. Given that the mean length of time between the visits was over 4 years, it is possible that the true underlying values of other variables in Table 2 have also changed. Hence some of these correction factors may be over-estimates of the correction factor required to obtain the association with the true error-free level at baseline. See Frost and White^16^ for further discussion of the impact of changes across the life course on the effects of measurement error and their correction.

For variables with two measurements taken per visit, a slight decrease in the correction factor can be seen when using the average of those rather than a single measurement. As expected, using an average of two error-prone measurements slightly decreases the measurement error.

## Conclusions

Measurement error in a continuous exposure in a univariable linear regression model leads to regression dilution bias. Repeat data taken on a sub-sample of participants from the main study can be used to calculate a correction factor for the regression coefficient. We have described how researchers can calculate the sample size for a repeatability sub-study, and given estimates of correction factors from UK Biobank data to help inform this calculation.

## Funding

The work for this paper was undertaken as part of the International Project on Cardiovascular Disease in Russia (IPCDR) project which was funded by a Wellcome Trust Strategic Award [100217], the Arctic University of Norway, UiT in Tromsø; the Norwegian Institute of Public Health; the Norwegian Ministry of Health and Social Affairs.
